# Review: Are we stumbling in our quest to find the best predictor? Over-optimism in sensor-based models for predicting falls in older adults

**DOI:** 10.1049/htl.2015.0019

**Published:** 2015-08-03

**Authors:** Tal Shany, Kejia Wang, Ying Liu, Nigel H. Lovell, Stephen J. Redmond

**Affiliations:** Graduate School of Biomedical Engineering, University of New South Wales, Sydney 2052, Australia

**Keywords:** geriatrics, biomechanics, body sensor networks, feature extraction, medical signal processing, feature selection, patient monitoring, reviews, telemedicine, review, sensor-based models, fall prediction, older adults, fall risk testing, wearable sensors, feature extraction, statistical models, cross-validation technique, feature selection, prognostic tools

## Abstract

The field of fall risk testing using wearable sensors is bustling with activity. In this Letter, the authors review publications which incorporated features extracted from sensor signals into statistical models intended to estimate fall risk or predict falls in older people. A review of these studies raises concerns that this body of literature is presenting over-optimistic results in light of small sample sizes, questionable modelling decisions and problematic validation methodologies (e.g. inherent problems with the overly-popular cross-validation technique, lack of external validation). There seem to be substantial issues in the feature selection process, whereby researchers select features before modelling begins based on their relation to the target, and either perform no validation or test the models on the same data used for their training. This, together with potential issues related to the large number of features and their correlations, inevitably leads to models with inflated accuracy that are unlikely to maintain their reported performance during everyday use in relevant populations. Indeed, the availability of rich sensor data and many analytical options provides intellectual and creative freedom for researchers, but should be treated with caution, and such pitfalls must be avoided if we desire to create generalisable prognostic tools of any clinical value.

## 1 Introduction

Falls experienced by older people lead to substantial morbidity and mortality, and have been identified as a significant public health issue worldwide [1]. As a result, and due to global ageing trends [2], fall prevention is considered vital. Various forms of fall prevention have been shown to be effective in the community, from home/group exercise, to multi-faceted programmes that address a range of fall risk factors [3]. These costly interventions can handle only limited patient volumes, as is also the case with dedicated fall clinics and their specialised equipment and personnel. There is thus a need, across a range of health care sectors, to correctly detect people at risk of falling in the wider population, and identify their fall risk factors, in order to allocate fall prevention measures in an accurate and timely manner. Many clinical tools have been developed for this purpose, from subjective, questionnaire- or observation-based tools with limited and often irreproducible prognostic value [4]; to advanced, objective balance and mobility assessments. The latter may be considered a gold standard measurement, but have limited value due to their one-off performance and often time- and resource-consuming nature [5]. There is a growing interest in an accessible, inexpensive approach that is simple to use and removes the subjectivity of many fall prediction or fall risk assessment tools. Ideally, such an approach would also accommodate regular repeat testing or even long-term monitoring.

## 2 Wearable sensors for fall risk testing

The use of miniature body-worn sensors for mobility-related monitoring has been on the rise, and has especially caught the public eye in relation to fall detection alarms [6]. Another area of use for wearable sensors, mostly accelerometers and/or gyroscopes, has been sensor-based fall risk testing (SFRT), which refers to fall risk screening or assessment [7]. These sensors are portable, inexpensive, and can continuously measure the movement of the wearer, both in a clinic or out in the real-world environment. The real-world option is becoming increasingly sought after due to its inherent flexibility and the avoidance of a potential Hawthorne effect, though real-world implementation might entail some loss of control over issues such as correct and consistent device placement, as well as the exact type of movement recorded at every stage (e.g. walking on level surface against stair negotiation) [8–10].

The signals recorded by a wearable sensor system supply a vast number of movement-describing quantitative variables, or features. This detailed quantification of movement would not have been achievable without the use of such sensors; or at most, may have been subjectively approximated through observation, thereby requiring supervised testing by qualified personnel. Researchers currently use the signal-derived features to create algorithms and mathematical models, with the aim of predicting future fall occurrence or classifying people into fall risk categories.

## 3 Current status of SFRT literature

The landscape of SFRT literature involving modelling (Table 1) consists of a diverse collection of quantitative models constructed on a myriad of different features, trained on different subject cohorts, and achieving different accuracy levels. In a review of 40 studies published prior to 2013, Howcroft *et al.* [11] highlighted notable sensor-based features, the model types considered, the validation processes employed and overall model performance. The authors stressed the problematic fact that most studies used fall history (FH), clinical assessment tools, or a combination of both as their standard (30, 32.5 and 22.5% of the studies, respectively), whereas the preferable option of prospective fall (PF) data (i.e. data of real falls occurring subsequent to the fall risk assessment) was only utilised in 15% of the studies [11].

**Table 1:** SFRT modelling studies, sorted by year of publication

A	B	C	D	E	F	G	H	I
Author, year	Final sample size	Model standard	Specific model targets	Features; (sensor features)	Potential violation of best modelling practice	Modelling	Maximum accuracy for entire cohort	Validation
Maki, 1997 [13]	75	PF (1y)	no falls: 32; 1 + falls: 43 (74 falls)	16; (10)		binary logistic regression, forward stepwise	73%	leave-one-out CV
Hausdorff, 2001 [14]	52	PF (1y)	no falls: 32; 1 + falls: 20	?; (4)	used only features that differed significantly, based on all data, between fallers/non-fallers	binary logistic regression per feature		resubstitution
Laessoe, 2007 [15]	94	PF (1y)	no falls: 80; 1 + falls: 14	9; (2)		scores rounded to 0-10; binary logistic regression per feature and for the test battery, backward stepwise	44%	resubstitution
Giansanti, 2008 [16]	60 for training; 200 for testing	POMA [17]	training only: low risk: 30; high risk: 30	(2)	multiple comparisons of classifiers across a series of papers using the same dataset; very high accuracies imply test data possibly used in model selection	neural network based on the Mahalanobis distance; other model types tested had reduced success	97%	stratified holdout
Kojima, 2008 [18]	153 with FH and fast walk data	FH (1y)	0/1 fall: 131; 2 + falls: 22	7; (2)	selected only two features (one sensor-based) that differed significantly, using all data, between fallers/non-fallers and had a low correlation with each other	canonical discriminant analysis	62%	resubstitution
Gietzelt, 2009 [19]	110	STRATIFY [20]; Barthel [21]	no risk: 20; fall risk: 90	(4)	authors noted when modelling STRATIFY: ‘elimination of all redundant gait parameters’ (no further explanation was provided)	decision tree; curve matching; linear median squared linear regression	90.5%	ten-fold or leave-one-out CV
Marschollek, 2009 [22]	110	PF (while in hospital)	no falls: 84; 1 + falls: 26	27; (6)		decision tree	90%	resubstitution
Greene, 2010 [23]	349	FH (5y)	no falls: 142; 1 + falls: 207	(44)	performed multiple comparison testing using all data before model selection and retained only significant features	binary logistic regression (three models: males, females <75 years old, females ≥75 years old)	77% (mean for the three models)	stratified holdout (80:20 split × 10 times)
Narayanan, 2010 [24]	68			54; (51)	feature selection performed outside of CV loop	linear least squares regression, sequential forward floating feature search		leave-one-out CV
Liu, 2011 [26]		PPA [25]	N/A	126; (123)				
Liu, 2011 [27]	68	FH (1y)	0/1 fall: 59; 2 + falls: 9 (36 falls)	126; (123)	feature selection performed outside of CV loop	linear discriminant classifier, sequential forward floating feature search	97% (71% for fall counts)	leave-one-out CV
Paterson, 2011 [28]	97 women	PF (1y)	no falls: 43; 1 fall: 25; 2 + falls: 29	16? (2?)	removed two non-sensor features that violated multicollinearity	binary logistic regression	67% for fallers/non-fallers	resubstitution
Bautmans, 2011 [29]	81	FH (6m), TUG [30] and POMA	old controls (no risk): 41; fall risk: 40	(6)		binary logistic regression, stepwise (forward likelihood ratio)	78%	resubstitution
Weiss, 2011 [31]	41	FH (1y)	0/1 fall: 18; 2 + falls: 23	30?; (27)		binary logistic regression, forward stepwise	88%	resubstitution
Marschollek, 2011 [32]	46	PF (1y, one phone call at end of 1y)	no falls: 27; 1 + falls: 19	15; (14)	wrapper feature selection, using all data, was used to select significant features prior to modelling; receiver-operator characteristic analysis likely performed post-hoc using all data	decision tree with regression; binary logistic regression	80%	ten-fold CV (×10 times)
Caby, 2011 [33]	20	FH (1y) and additional clinical input	no risk: 5; fall risk: 15	(67)	various feature pools and algorithms investigated outside CV loop; pre-selection of features performed using Holm-corrected multiple comparisons; unclear if feature selection done using training data only during CV	neural network; support vector machine; *K*-nearest neighbour; naive Bayes classifier	100%	leave-one-out CV
Senden, 2012 [34]	100	POMA	low risk: 50; high risk: 50	13?; (9)	removed two sensor features with low correlation with the target	linear regression		resubstitution
Fuke, 2012 [35]	17	BBS [36]	no risk: 8; some risk: 4; high risk: 5	(4)	data was scaled prior to modelling using all data	support vector machine		leave-one-out CV
Greene, 2012 [37]	120	FH (5y)	no falls: 55; 1 + (serious) falls: 65	(44)	single final list of features for CV ( × 10 times) implies feature selection was performed outside of CV loop; multiple feature subsets explored outside of main CV loop; confidence intervals reported when CV results not i.i.d.	support vector machine using default values for the hyperparameters, sequential forward feature selection	72%	ten-fold CV ( × 10 times)
Greene, 2012 [38]	226	PF (∼2y, one phone call at end of 2y)	no falls: 143; 1 + falls: 83 (144 falls)	(44)	single final list of features for CV ( × 10 times) implies feature selection was performed outside of CV loop; confidence intervals reported when CV results not i.i.d.	regularised discriminant classifier models (for males, females <75 years old or ≥75 years old), sequential forward feature selection	80% (mean for the three models)	ten-fold CV ( × 10 times)
Schwesig, 2012 [39]	141	PF (1y, nursing home records)	0–2 falls: 124 3 + falls: 17 (171 falls)	12?; (6)	confidence intervals appear to be reported using the same test data as used for model fitting	binary logistic regression, backward stepwise		resubstitution
Doheny, 2013 [40]	39	FH (5y)	no falls: 20; 1 + (serious) falls: 19	(70)	removed features with low reliability (≤0.7) after test–retest using all data; feature selection performed outside of CV loop; confidence intervals reported when CV results not i.i.d.	binary logistic regression, sequential forward feature selection	74%	leave-one-out CV
Riva, 2013 [41]	131	FH (1y)	no falls: 89; 1 + falls: 42	27 + ; (24)	factor analysis performed using all data to identify salient sensor features prior to multivariate model selection	binary logistic regression (univariate and multivariate), forward stepwise)	72.5%	resubstitution
Doi, 2013 [42]	73	PF (1y)	no falls: 57; 1 + falls: 16	10 + ; (6)	using all data, pre-selected significant features using multiple comparisons against the faller/non-faller target	binary logistic regression, forward stepwise		resubstitution
Weiss, 2013 [8]	71	FH (1y)	0/1 fall: 39; 2 + falls: 32	26?; (19?)	using all data, removed features that did not meet corrected significance based on FH, and features with ≥0.8 correlation	binary logistic regression, backward stepwise	72% FH;	resubstitution
		PF (6m)	0/1 fall: 59; 2 + falls: 12				95% PF	
Greene, 2014 [43]	124	FH (1y)	no falls: 76; 1 + falls: 48	(150?)	unclear if feature selection done using only training data inside CV loop; confidence intervals reported when CV results not i.i.d.	support vector machine, sequential forward feature selection	83%	stratified ten-fold CV ( × 10 times)
Rispens, 2014 [10]	110	FH (1y)	0/1 fall: ?; 2 + falls:(? falls)	(57)	pre-selection of features using multiple significance and reliability tests on all data prior to modelling	negative binomial regression for number of falls; binary logistic regression for multiple fallers against 0–1 fall		resubstitution
Liu, 2014 [44]	98	PF (1y)	no falls: 50; 1 fall: 28; 2 + falls: 20	125; (123)	several different classification tasks (fallers, or multiple-fallers) and various different starting feature subsets evaluated	binary logistic regression, forward stepwise	83% for one of the multiple-faller models	two-fold CV
Schwenk, 2014 [45]	77	PF (3m, diaries, but dementia patients)	no falls: 49; 1 + falls: 28	20; (9)	pre-selection of features using multiple univariate significance tests, using all data, prior to modelling; four different feature subsets/models investigated	binary logistic regression (univariate and multivariate), stepwise		resubstitution
Gietzelt, 2014 [46]	38 (2m); 33 (4m); 28 (8m)	PF (max 8m, nursing home records)	no falls: 15?; 1 + falls: 13 (26 falls)	?; (12?)	correlation-based pre-selection of features using all data; specific trained models are shown, even though ten-fold CV should result in ten models per task	decision tree	88.5% (8m)	ten-fold CV
Van Schooten, 2015 [9]	169	FH (6m)	no falls: 109; 1 + falls: 60	67?; (33?) ( + 26 sensor features interactions)	features were transformed to *z*-scores using all data prior to modelling; pre-selection of features using multiple significance and reliability tests on all data prior to modelling; multiple potential models explored and best reported	binary logistic regression per feature and also in various combinations, forward stepwise		resubstitution
		PF (6m)	no falls: 110; 1 + falls: 59					

Column B specifies the final number of subjects, excluding sub-cohorts of young controls. Rows highlighted in grey are studies that used prospective falls (PF) as the basis for the dependent variable; other studies used fall history (FH), clinical risk assessments, or various combinations, as noted under column C. Column C also contains the fall reporting period in parenthesis (i.e. 1y = one year, 6m = six months); fall data was recorded using self-reporting diaries, sometimes accompanied by phone calls, unless otherwise stated in this column. Column D includes the model targets, and pertains to the number of individuals in each category (i.e. fall risk level or sub-group based on number of falls); where available, the actual number of reported falls within the entire cohort is included as well, even if not used for analysis. Column E contains the number of features available for analysis, with the specific number of ‘sensor-extracted’ features in parentheses. In certain cases, only sensor features were used, or it was not possible to accurately report the overall number of model features. Under column I, CV stands for cross-validation. Empty fields in the table imply that the specific aspect was not mentioned in the publication, or could not be confidently deduced from the provided information. A ‘?’ symbol appears where there might be such uncertainty regarding the specific number(s) of model targets and/or features.

Recently, Palumbo *et al.* [12] conducted an insightful probability modelling exercise, using analytical evidence to calculate theoretical estimates for the maximal prognostic abilities of an ideal fall prediction tool. This exercise was based on the use of actual fall data from four different populations (all from Australia, New Zealand and USA), accompanied by certain assumptions regarding fall rates and their distribution. The results indicate that the maximal accuracy of a fall prediction model, attempting to identify people with at least one fall incident over the course of a year from the starting point (i.e. from the time of the testing) would not exceed 0.81, with a maximal area under the curve of 0.89. The results differ slightly for shorter or longer follow-up periods and for models intended to identify people who fall multiple times rather than those who fall only once during the prediction period. This report is instrumental as it forces us to examine and question the boundaries of what we should expect to achieve in the challenging task of predicting falls.

Returning to Howcroft's review, which presented accuracy levels ranging from 62 to 100%, one must ask how some of the models achieved greater accuracy than Palumbo's suggested upper limit. Howcroft *et al.* touched upon the limited validity of models where ‘validation’ was performed only on the training data, thereby inflating the model's accuracy. This is indeed an issue that plagues the published SFRT literature. The aim of the current work is to further elaborate on concerns involving the statistical methodologies that have been employed across the SFRT literature, and which carry the very probable risk of generating models that will only ever work on the data used for their training.

## 4 Summary of Table 1

The past two decades have been witness to a constant rise in SFRT research and relevant publications. In this review, we chose to focus on important aspects which apply only in studies that employed modelling analyses, and include, for example, methods of feature selection, the types of models used and validation strategies (or lack thereof in some cases). A choice was made to include only a single performance parameter per study in column H of Table 1 (maximum accuracy for the entire study cohort). This was done for consistency between the studies and for the reader's convenience; however, it is acknowledged that this could mask certain cases of unbalanced sensitivity and specificity outcomes. Table 1 also does not include aspects such as subject demographics, setting type (community, hospital, aged care facility), cohort characteristics, movement type(s), recording period, sensor(s) and their location in order to focus on matters pertinent to modelling and validation.

Model types used in SFRT research have predominantly been drawn from either the regression family (multiple linear regression to model against a numerical fall risk score, logistic regression, discriminant function analysis, negative binomial regression to model fall incidence), or the pattern classification family (support vector machines, artificial neural networks, decision trees). Feature extraction is a meticulous process, and as such, is often not sufficiently detailed in published papers. The chosen features, or the manner in which their extraction was achieved, often differ between studies. The number of features incorporated into each model also varied substantially between studies, as did the reported model performance – with accuracy levels as low as 44% [15] to as high as 100% [33]. This range is larger than that reported by Howcroft *et al.* (62–100%) [11], since it encompasses several studies that were not included in their review. The remarkably successful performance of several fall risk classifiers and estimator models in the SFRT literature should raise concern, particularly in the case of prognostic tools reporting expected near perfect accuracy. This issue and others will be addressed in the following sections.

## 5 Issue 1: publication bias

First and foremost, the persistence of publication bias is pervasive through all of scientific research [47]. In our context, better performing models may have a greater chance of publication due to our eagerness to reward those efforts which appear to have been successful in creating accurate prediction models. Unfortunately, this universal problem in research still lacks a solution, and remains an issue that we must be mindful of while carrying on. It is especially exacerbated through the use of small sample sizes, where confidence intervals are large and false discoveries become more likely. Any bias on the part of the researchers (intentional or not) amplifies the rate of false discovery [47]; these biases often arise through the use of inappropriate modelling approaches (discussed in detail later), or selective or distorted reporting, which can easily go undetected.

## 6 Issue 2: inadequate sample size

The power of a study (the probability of detecting an effect when one truly exists) is dependent on both the effect size and the number of samples acquired to detect that effect. The effect size in fall prediction studies is relatively small. If we achieve Palumbo *et al.*’s estimated theoretical maximum of 81% accuracy in fall prediction, compared with 56% when guessing (assuming one in three people fall, 56% = (33%)^2^ + (67%)^2^), we obtain a Cohen's kappa statistic of 0.57 = (0.81–0.56)/(1.0–0.56) as a theoretical maximum, which is defined as only a moderate agreement, or we could say, a moderate effect size. Therefore, the statistical effect we are looking for which allows us to predict falls is not a large one, so we should expect many research studies in this area to report negative results, especially if the sample size is small.

As can be observed in Table 1, sample sizes in fall studies are often unsatisfyingly small. While clinical trials in medicine commonly exceed thousands of patients, many SFRT studies included fewer than a hundred participants, with further patient or data loss due to premature termination (illness, death, withdrawn consent), non-compliance during unsupervised recordings, inability to perform certain movements, or technical reasons involving the sensor systems or signals. One of the biggest hindrances for this research area, which limits sample size, is the long and intensive follow-up period. Currently, the ideal design is to record fall events using self-reporting diaries over a 1-year period. This follow-up can be an expensive undertaking when it involves research staff persistently contacting hundreds of participants to request they return their weekly/monthly fall diaries, as well as verify or elaborate on the reported information in case of a fall.

However, it is not just total sample size that we need to be concerned about to ensure sufficient study power. When a study is conducted where the dependent variable is the number of falls experienced by each subject, the raw number of events needs to be sufficiently large to avoid small-sample bias [48]. Samples where the number of positive events is very few relative to total sample size may give rise to distorted models [49]. Falls may prove to be a relatively rare event, especially for moderately healthy older cohorts. Laessoe *et al.*, for example, reported only 14 subjects out of 94 who experienced at least one fall during follow-up, and only four subjects who reported recurrent falls [15].

## 7 Issue 3: misuse or lack of model validation

This section discusses some issues related to model validation which are common throughout all disciplines relying on learning algorithms to infer the structure of experimental data.

Throughout this section, when the term ‘training’ is used, it implies the entire model selection and fitting procedure. This is a critically important point. Model selection relates to all steps involved in training a model which is to be later evaluated, including: (i) selecting which features should be included; (ii) choosing which specific pattern recognition model should be used; (iii) choosing the complexity/structure of the classifier model, if there is such an option; and (iv) final tuning of the model hyperparameters. In completing steps (i)–(iv), the test data should *never* be used. There is more to say on this topic of feature and model selection later, but first we discuss various model validation procedures and their relative advantages and disadvantages, assuming that model specification and training is done correctly throughout.

### 7.1 Resubstitution

Possibly the worst type of validation which can be performed is called resubstitution. The model is trained using all of the data and then validated by resubstituting the same data as test data. It is obvious that a sufficiently complex model could fit the training data (and in this scenario also the test data) such that perfect performance can be achieved. This method is never recommended unless some strict regularisation methods are applied to the model in order to constrain its flexibility and reduce the likelihood of overfitting.

### 7.2 Holdout

A common approach to model validation is to use a holdout technique. The data is randomly split into training and testing groups (often in the size ratio of 2/3 and 1/3, respectively). If there is a known confounding issue, such as the cohort consisting of both able and extremely frail individuals, this information can be used to balance the groups through a process known as stratification. The model is then trained with the training data and validated using the testing data. This approach has some very appealing statistical properties; the most important of which stems from the fact that the test data is completely independent and identically distributed (i.i.d.), allowing confidence intervals to be calculated for many common performance metrics, provided the trained model is the final model to be used in the wild. If the model is then retrained before deployment, using all of the available data, these confidence intervals do not capture the sensitivity and the stability of the algorithm to changes in the training data, and could result in much worse generalised performance than was estimated. This sensitivity of models to changes in training data is an open area of research in the pattern recognition field [50].

A criticism of the holdout technique, particularly for small datasets, is that it is wasteful or inefficient. The holdout dataset used for testing is small, resulting in large confidence intervals; the training set is also relatively small resulting in weaker models (often referred to as a ‘negative bias’ in the estimated results). The influence of randomly partitioning the data into training and testing sets introduces an additional variance into model training, which is exacerbated if the learning algorithm is not stable, as discussed above. The inefficiencies of holdout methods have inspired the cross-validation (CV) approach to model validation, but as we will discuss below, CV estimates can have a very large variance which is difficult to estimate [51], and it is still unknown under what conditions (sample size, choice of learning algorithm etc.) CV can theoretically give better estimates of future model performance compared with simple holdout validation [50].

### 7.3 Cross-validation

CV involves partitioning the *N* data samples into *K* non-overlapping subsets, often called folds. The model is trained using *K*−1 of these folds, and tested with the remaining fold. Training and testing is repeated *K* times, so that the data in each fold is tested once. The final accuracy is reported as the testing accuracy over the *N* test samples. The choice of *K* is typically chosen to be *K* = 5 or *K* = 10 on purely empirical grounds [52], although *K* = *N*, called leave-one-out CV, is commonly used. There are other similar techniques, such as bootstrapping or jackknifing, but they operate in a similar manner.

CV has become a pervasive technique through all of machine learning for estimating generalised model performance, despite its statistical properties being very poorly understood [50, 51]. This is extremely worrying, given its use to inform decisions in biomedical fields. More recently, deeper analyses of this validation method have uncovered some unsavoury properties, particularly for smaller datasets [53] and those with outliers [51], which raise questions over whether there is benefit in using CV as a performance estimator at all. In particular, we do not know yet how to estimate the variance of CV estimates and hence calculate confidence intervals [51]. The primary problem here is that the test results derived from each test fold will at some point be part of *K*−1 other training data subsets, and this interdependence precludes the use of the central limit theorem to estimate confidence intervals, as the inter-fold errors are not i.i.d. This result should be emphasised: the use of CV is widespread in many disciplines, but we have no way to reliably appraise the precision of the resulting performance estimates, which can be very poor if the sample size is small. Occasionally, authors list the variance of results between CV folds as a measure of confidence in the mean result, but this has not been theoretically shown to be a meaningful measure of the variance/precision of the mean CV result, and in practice can overestimate confidence in the future performance of the model [51].

Furthermore, to repeat the important point stated earlier, it is not theoretically known (for all learning algorithms) whether CV gives a better estimate of future model performance than a simple holdout validation method, especially for datasets with small sample sizes [50, 51, 53]. This is somewhat ironic, since the unavailability of additional validation data is the primary motivation for using CV in the first place.

### 7.4 Repeated CV

As stated above, there is an additional variance introduced in CV due to how the data are randomly partitioned into training and testing sets, and some have tried to average this effect away using repeated CV; that is, repeating the partitioning and CV processes multiple times with the same data. However, it has been shown that the more serious effects discussed above, of inter-fold dependence of data and the negative bias introduced by only training on *K*−1 folds (especially if the learning algorithm is unstable), are more dominant confounders, and this repeated CV approach is probably a waste of computational resources and time [51, 54].

The point to be made in all of this is that we should be wary of the results reported using CV, and perhaps even prefer results obtained using a wasteful holdout method for which the margin of error is easily calculable (assuming the trained model is the final model to be used in the future), although this margin may be unacceptably wide for small sample sizes. Independent external validation of the final model in a separate study with a large sample size would be ideal. One might argue that there is no distinction between using a holdout method and performing an external validation (that is, repeating the entire study), but this is not true for several reasons. First, performing an external validation using different testers and cohorts can uncover experimental biases. Second, as will be discussed later, the model training process is fraught with temptation and opportunity to overfit the available data – how many reported research results were the outcome of a single modelling attempt, where the researcher has not tweaked the model parameters at least once to improve the result? External validation removes this temptation.

With this background in place, we can return to the discussion of validation of specific fall prediction models reported in the literature. Many SFRT studies noted that their performance reports were based on resubstitution, which could result in over-optimistic estimates of model performance if the model training process is not sufficiently constrained. Some papers did not mention model validation at all, which leads to the assumption that they also utilised resubstitution, and hence were categorised as such in Table 1. Other studies used various forms of CV, which carries a range of implications, as discussed above. It seems that only Giansanti *et al.* [16] and Liu *et al.* [44] employed testing and training on clearly separate datasets. The very high accuracy of Giansanti's study [16] implies that the test data was possibly introduced during the training phase, perhaps through reuse of the same dataset across a series of publications [16, 55, 56], through additional feature and model evaluation which was not reported, or simply because the performance oriented mobility assessment (POMA) score is easier to estimate than falls are to predict (falls are not used in their modelling).

Very few authors provide any measure of confidence in their results. The difficulties in calculating any confidence intervals for the holdout method (when the model would be retrained before deployment), or whenever CV is employed, have already been discussed above. Among the few articles which do report confidence measures, Greene *et al*. [37, 38, 43] reported confidence intervals from repeated CV; however, as discussed earlier, such measures have not been shown to be reliable measures of the true variance of CV. In general, estimating confidence intervals for CV results is a difficult problem that has yet to be conclusively solved [50].

To summarise this section, the main concern arising from this review is the complete lack of external validation across the SFRT literature. In order of preference, external validation is preferred first, followed by holdout validation (listing confidence intervals for testing), then CV. Resubstitution validation or complete omission of model testing is not helpful and should not be published (this includes standard results of stepwise regression performed by statistical packages). Researchers in the SFRT field could learn from previous attempts to develop non-sensor-based fall risk assessment tools, which have had their own trouble with external validation, in that some have not been validated properly, while others have performed poorly in external validation studies (i.e. when conducted in a different subject group and/or a new site, and sometimes also by authors other than the original tool developers) [57, 58]. It would be best to establish early on whether SFRT is also handicapped by this aspect, or whether the greater measurement objectivity offered by the use of sensors would be able to overcome certain differences between subject cohorts, sites and experimenters, which seem to stand in the way of establishing the generalisability of many prognostic fall risk tools.

## 8 Issue 4: the curse of dimensionality

This section deals with the problem of model selection. This problem can be further divided into two issues which are distinct yet seemingly intertwined. The first issue relates to the problem of selecting an appropriate model (selecting features, choosing the model type and structure, setting hyperparameters) when there is a finite pool of data available to do so. The second issue relates to the (now all too common) abuse and misuse of machine learning techniques, usually in an attempt to guide model selection in the face of too few data from which to learn the best choice of model.

Selecting a model involves deciding which raw data will be analysed, which features will be extracted from these data, which learning algorithm will be used, and specifying any options available within this algorithm, such as setting and thresholds or hyperparameters. The ‘curse of dimensionality’ is a phrase which captures the sense that there are infinitely many ways to extract features from signals and an endless list of learning algorithms, and that this gluttony of choice is unhelpful when there is only a finite pool of data upon which to base a final decision, when the hope is that the model will later perform well during everyday use in other relevant cohorts.

To frame this in the context of SFRT, the richness of inertial sensor data entices researchers into exploring as many feature sets and processing techniques as we find interesting. Some researchers combine signal features with other potential predictor or confounding variables, such as demographics (age, gender, weight, height, past falls) or results of other assessment tools. The consequence of this is that the model building process begins with a very large pool of features from which we must find the most potent of predictors to construct the model(s), or equivalently, having a low ratio between number of events and number of selectable features available at the model development stage [37]. Examples of this in the literature include the considerable number of covariates, particularly sensor features, in the studies reported by Narayanan *et al.* [24], Liu *et al.* [26, 27, 44], Doheny *et al.* [40] and Greene *et al.* [37, 43].

Given a limited sample size, the first realistic solution is to try to reduce the size of the feature pool prior to model building as sensibly as possible. Ideally, knowledge from earlier research should inform this cull, resulting in a smaller number of features that are more likely predictive, but this approach is confounded by the fact that earlier research is also affected by the same problems discussed throughout this Letter. Researchers certainly strive to derive meaningful features from the sensor signals by incorporating a priori information regarding the nature of gait, for example, in an attempt to maintain clinical relevance [8, 42]. This approach should be encouraged as it could resolve some of the issues outlined in this Letter.

All remaining methods used for model selection, different to those that use external a priori knowledge, are driven solely by an analysis of the available data. Echoing the previous section on validation, in what follows the most important point to be made is that the testing data (both features and targets) should *never* be used to inform the model selection procedure. Notably, feature selection is sometimes considered a procedure which is external to model selection, but this is a fallacy; the act of feature selection can simply be considered as setting binary model hyperparameters, and therefore relates to model selection.

If a holdout validation method is used, only the training set should be used for model selection, including feature selection. This principle holds also for CV, except this entire model selection process will be repeated *K* times, for each of the *K* folds. This will result in *K* sets of model parameters (features, hyperparameters etc.), making it difficult to report a single preferred model without performing an additional final model selection using all of the data once the usual CV process to estimate future performance is complete. Another consequence of using CV to estimate future model performance is that it is often inconvenient for authors to publish the full details of all the *K* feature subsets that were chosen, which is understandable but undesirable as it limits the extent to which future work can build on past advances. The supply of such details in attached appendices or as histogram plots where applicable may be beneficial for pre-selecting features for future research, or understanding model stability.

It should also be noted here that CV is often used again as part of the model selection process, as it provides a reasonably strict way to evaluate the usefulness of a potential feature or model parameter. This should not be confused with the use of CV to estimate generalised future performance of a specific selected model, discussed in the previous section. This second CV loop, used to select features and other model parameters, is nested within the outer validation loop and operates only on the training data, subdividing it into a further *K* folds. Should holdout validation be used instead of CV, there is only one training set for this CV-based model selection procedure to operate on, so the picture is clearer, and a single selected predictors set may be published; however, it may be difficult to know how stable the algorithm will be if retrained by another research group on new data.

An alternate approach to feature selection, called stepwise regression (linear, logistic, binary logistic etc.), is implemented by many common statistics packages [59]. This stepwise feature selection method performs *t*-tests on model coefficients after a potential model is trained using a subset of selected features (often called predictors). The outcomes of these *t*-tests guide the inclusion or removal of features until no further features meet the criteria for inclusion. There are two potential pitfalls with the use of this method. First, if a sufficient number of features are available for consideration, chance agreement between the modelling target and the potential predictor may lead to its inclusion (this relates to the standard multiple comparisons problem), prompting the use of stricter thresholds on the *t*-test *p*-values for feature inclusion. The second pitfall relates to validation. The classification results reported by many software packages for the final model are the result of resubstitution-based validation, and hence very likely overly-optimistic, as the test data has not been held separate from the training data. If a statistics software package has the option to perform CV, this should be done, or preferably test data should be separated before modelling begins to allow holdout validation later.

Note also, on the issue of separating training and test data, even if a method similar to principal component analysis, which is ignorant of the target values (e.g. classification sub-groups or fall counts), is used to reduce the dimensionality of the feature pool prior to model selection, the data transformation should only be learned from the training set and then later applied to the testing set.

For the interested reader, some common pitfalls of model selection and validation in pattern recognition were bemoaned in a very clear way some 20 years ago by Chatfield [60], and again more recently by Nowotny [61], indicating that the issue has not improved.

Given what is presented above, it should be obvious that (especially for the field of SFRT research, which has at most several hundred learning examples) including a large number of features at the outset, or a learning algorithm with great flexibility, or a learning algorithm which is not stable, will result in a model choice which is highly unlikely to generalise well. We now discuss the SFRT literature in relation to these issues.

As can be seen in column F of Table 1, the SFRT literature displays varied approaches to the issue of feature pre-selection, prior to their use in model construction and validation. The use of approaches termed ‘exploratory analysis’ or ‘preliminary analysis’ which use all data to pre-select features for modelling should raise our level of concern, as features should only be selected from the training data and done so independent of the test data. Pre-selection was sometimes done by omitting features that did not achieve a desired level of statistical significance when compared between the subject sub-groups or when mapped individually to the dependent variable using all of the available data. As explained above, this is advised against; moreover, making multiple comparisons between every potential predictor and the dependent variable dramatically increases the chance of a type-I error occurring [62]. As mentioned earlier, this risk associated with performing multiple comparisons is also true for stepwise regression methods if proper correction of inclusion thresholds is not applied. Only very few groups attempted to address the problem of multiple comparisons, in general, by using lower *p*-values or known adjustments, such as the Holm correction, the Hochberg technique or Bonferroni [8, 28, 31, 33].

Another approach to reducing the size of the feature pool involves the removal of features that correlated with each other by more than a certain percentage, under the assumption that high inter-correlation suggests a degree of redundancy. Weiss *et al.* [8] noted removing features with greater than 80% inter-correlation, while others simply noted using features with low inter-correlation [18], removing features that violated multicollinearity [28], using correlation-based filtering [46], or removing ‘redundant gait parameters’ (without further clarification) [19]. In the absence of sufficient amounts of data, this is a valid method to select potential predictors with a much lessened risk of the selected model overfitting the training data; although we now risk missing the effect if a salient feature is erroneously discarded in this process which purposely ignores modelling target values. Again, it is important that only the correlations among the training data be used in this operation, and the test data must be kept safely separated. Another method involved the removal of sensor features with low intra-class correlations in reliability testing (test–retest) [63], an issue which is worthy of a separate discussion. Most studies, however, did not address or resolve possible feature correlations or their reliability.

Many groups did not attempt to reduce their initial feature pool at all, and instead utilised various forms of stepwise selection or stepwise regression in their model building processes. In some cases, it was difficult or impossible to accurately deduce the number of features, including sensor features that were introduced to the model.

In many cases, as itemised in column F of Table 1, proper separation of training and testing data appears not to have been maintained, or other potential violations in best modelling practice have occurred (again, see [61] or [60] for recommendations), most likely resulting in inflation of results.

Assuming that model selection is performed in a valid way, such that no aspect of the testing data can influence the model choice, it is still known to be a very difficult task to select a model which does not overfit the training data, particularly if there are few training examples available, and more so if there are many potential predictors and model parameters to choose from. Overfitting often results in poor performance of the selected model during subsequent validation, and is clearly something to be avoided. As was stated earlier, CV is often used to assess potential model options (using only the training data) before a preferred model is selected for validation. However, we know that CV can have a large variance, especially if the model is unstable, so is it really a good way to guide our model selections? Research into model stability and risk is still ongoing in the machine learning field and to date there are no concrete guidelines to follow when sample sizes are small, as is commonly the case for SFRT. The approach used by stepwise regression of performing *t*-tests on model coefficients appears a more theoretically sound means of model selection, as an alternate to the empirical CV-based evaluation of potential models. However, stepwise regression is not immune from the curse of dimensionality either, and a substantial ratio of training examples to potential features must be maintained if modelling is to succeed. Again, as for machine learning algorithms, how substantial this ratio should be is not known with certainty.

Unsurprisingly, as the theoretical work continues, several heuristics have been suggested to promote the generalisation capabilities of multivariate models, such as limiting the number of predictors in a model to one per ten target outcome events (which may be fall incidents or number of individuals in the smaller sub-group being classified) to minimise bias in the regression coefficients. Others even suggest a stricter 1:20 ratio, though these recommendations may differ according to the model type being used [43, 44]. For example, it has been suggested that the 1:10 rule may actually be relaxed in the case of logistic regression [64], which is noteworthy given the prevalence of this model type in SFRT research. Nevertheless, it is of interest to refer to columns D and E of Table 1 and note that many studies were found to exceed the recommended 1:10 ratio of features to target events (or individuals), suggesting that overfitting is likely in many of the models presented.

## 9 Issue 5: nature of dependent variables

Finally, we discuss the issue of what it is we want to estimate or predict. For the studies reviewed here, modelling was performed in relation to past falls (also called fall history) or PFs (i.e. falls that occurred before or after the date of assessment, respectively); or clinical fall risk assessment tools, such as the Tinetti score (POMA); or a combination of these. Many of the presented studies did not incorporate a prospective design; however, looking at Table 1, a positive trend can be noticed towards more prospective studies in recent years (rows highlighted in grey).

Most studies did not analyse the fall events directly; instead, subjects were divided into groups, whether low risk against high risk, non-fallers against fallers or fallers against multiple fallers (unfortunately, using different definitions for these allocations across the studies). Various classifiers and binary logistic regression were then used for modelling. These methods reduce the potential resolution offered by the raw data, and remove the element of fall risk in relation to fall rate. One could argue, however, that classifying people into risk groups is more than sufficient in the context of fall risk screening, and that the use of raw fall counts may be redundant.

An additional issue worth mentioning involves the accuracy of the collected fall data. Fall records based on patient recollection have been shown to lack reliability [65]. Fall diaries or carer reports are currently the gold standard option for this type of data, but these records might also exclude some falls for various reasons (e.g. patients trying to hide problem severity, carers worried about being held responsible, falls gone unnoticed). Perhaps in the future, this aspect would improve via the continuous use of wearable sensors for fall detection.

With regards to FH being used as the standard, we should note that this might be associated with misleading results, stemming from changes in gait patterns and other movement adaptations, perhaps due to injury or a new-found fear of falling, which are directly linked to the past fall and manifested by the individual during the assessment.

The involvement of clinical assessment tools in SFRT research would remain valid, but not as targets for model training. Instead, the performance of SFRT tools (that are shown to be valid) should be compared with common clinical assessment tools to ascertain which performs better, or whether it would be in our interest to combine both approaches. This is related to the realisation that SFRT by itself is unlikely to capture all risk factors for falling.

## 10 Suggested steps forward

In an ideal situation, models should be built (or trained) on one sufficiently large dataset, then validated on another dataset, then externally validated multiple times across completely different samples of the same over-arching cohort of interest, with each person only ever meeting the model once [60]. This is not easy to achieve, particularly in the context of human-based fall research which carries many challenges. However, we should not allow insufficient criticism of our own analytical methodologies to become yet another major challenge of SFRT research.

Two ways to minimise small-sample bias can be suggested: (i) recruit a cohort large enough to ensure the number of fall incidents is sufficient; or (ii) collect data strategically, specifically to manoeuvre around this issue (e.g. by initially focusing on high risk cohorts such as patients with Parkinson's disease). The latter is only desirable when extremely large samples are infeasible to achieve the first option. To complement power calculations, it may be useful to consider the following when planning trial recruitment – based on the generally recommended 1:10 ratio of features to events [62], and the epidemiological finding that a third of all people over 65 years of age will fall at least once per year [1], we should recruit (at least) 30 individuals per planned predictor variable for a 1-year prospective trial. Of course, there are never guarantees: the final fall rate may be more or less than the expected 0.33/year, participants may drop out of the study, and some signal data may prove unusable; hence, a degree of flexibility is warranted.

Clearly, it is vital to manage the curse of dimensionality, whether by tightening the significance threshold, removing highly correlated (potentially redundant) features, choosing correct statistical techniques, performing appropriate validation where possible, or realistically, a combination of the above. Finding the right balance which allows us to consider as many potentially useful predictors as possible without bloating the probability of false positives is a fine balancing act indeed. Feature extraction should be conducted thoughtfully with full awareness of the limitations in whichever approach we take. Indeed, it is important to stay open-minded at the exploratory stage in order to avoid false negatives or the removal of features that could be predictive but were merely not detected as such.

Specifically regarding the popularity of CV, this family of validation methods became popular out of computer science and machine learning, as they appeared to enable model evaluation to occur with less data, so clearly the attraction is understandable. Their negative effects on the integrity of models pose an interesting problem as the full severity and extent of these problems are currently unknown. Thus, there is a gap here for both theoretical and evidence-based research to evaluate and quantify the limitations of CV processes in prediction analyses, from a statistical and mathematical basis. The findings would clearly be of significant value, not only to fall researchers but to the entire statistical modelling community.

## 11 Conclusion

The body of literature on sensor-based fall risk assessment and fall prediction studies is likely presenting an over-optimistic view of what is achievable in this field, given that most publications suffer from at least one if not several limitations, including small sample sizes, excessively large feature pools, model overfitting and lack of model validation, or misuse of modelling methods. The movement of research towards sensor-based fall prediction tools is important and encouraged, and given the potential of these systems to offer a simple, portable, inexpensive solution, it is likely to only increase in the near future. Hence, we owe it to our study participants, future patients and to ourselves to ensure that these fundamental issues are addressed in our efforts going forward.
